# Supplementary material for: Framework for rapid comparison of extracellular vesicle isolation methods
Source: eLife. 2021 Nov 16;10:e70725. doi: 10.7554/eLife.70725 (PMC8651285; doi:10.7554/eLife.70725)
Supplement: Transparent reporting form [file elife-70725-transrepform1.docx]

***eLife’s* transparent reporting form**

We encourage authors to provide detailed information *within their submission* to facilitate the interpretation and replication of experiments. Authors can upload supporting documentation to indicate the use of appropriate reporting guidelines for health-related research (see [EQUATOR Network](http://www.equator-network.org/%20)), life science research (see the [BioSharing Information Resource](https://biosharing.org/)), or the [ARRIVE guidelines](http://www.plosbiology.org/article/info:doi/10.1371/journal.pbio.1000412) for reporting work involving animal research. Where applicable, authors should refer to any relevant reporting standards documents in this form.

If you have any questions, please consult our Journal Policies and/or contact us: [editorial@elifesciences.org](mailto:editorial@elifesciences.org).

**Sample-size estimation**

- You should state whether an appropriate sample size was computed when the study was being designed
- You should state the statistical method of sample size computation and any required assumptions
- If no explicit power analysis was used, you should describe how you decided what sample (replicate) size (number) to use

Please outline where this information can be found within the submission (e.g., sections or figure legends), or explain why this information doesn’t apply to your submission:

We decided how many different conditions or samples to use based on how many samples we could run at one time on the HD-X machine to facilitate a direct comparison. For the experiment in Figure 6, we used 4 replicates of each column type for the experiment as that was how many samples we could load at one time.

**Replicates**

- You should report how often each experiment was performed
- You should include a definition of biological versus technical replication
- The data obtained should be provided and sufficient information should be provided to indicate the number of independent biological and/or technical replicates
- If you encountered any outliers, you should describe how these were handled
- Criteria for exclusion/inclusion of data should be clearly stated
- High-throughput sequence data should be uploaded before submission, with a private link for reviewers provided (these are available from both GEO and ArrayExpress)

Please outline where this information can be found within the submission (e.g., sections or figure legends), or explain why this information doesn’t apply to your submission:

For all Simoa measurements in the paper, all samples were run in duplicate. These are technical replicates. Coefficients of Variation for technical replicates for all experiments described in Figures 2-4 and Supplementary Figures S5&S6 are shown in Supplementary Figures S7. For the comparison experiment, ultracentrifugation and the ExoQuick kits were done on two separate days and the results were averaged. Variability of these methods between the different days is shown in Supplementary Figures S1. These experimental details are listed in the Materials and Methods sections. For Figure 6, each condition was run with four replicates of each column. This is described in the figure legend.

**Statistical reporting**

- Statistical analysis methods should be described and justified
- Raw data should be presented in figures whenever informative to do so (typically when N per group is less than 10)
- For each experiment, you should identify the statistical tests used, exact values of N, definitions of center, methods of multiple test correction, and dispersion and precision measures (e.g., mean, median, SD, SEM, confidence intervals; and, for the major substantive results, a measure of effect size (e.g., Pearson's r, Cohen's d)
- Report exact p-values wherever possible alongside the summary statistics and 95% confidence intervals. These should be reported for all key questions and not only when the p-value is less than 0.05.

Please outline where this information can be found within the submission (e.g., sections or figure legends), or explain why this information doesn’t apply to your submission:

For Figure 6, error bars represent standard deviations of the measurements of the four different replicates of each condition. This is indicated in the figure legend. For comparison, of SEC columns, we are looking for trends from a large number of samples to then choose best columns for Figure 6.

(For large datasets, or papers with a very large number of statistical tests, you may upload a single table file with tests, Ns, etc., with reference to sections in the manuscript.)

**Group allocation**

- Indicate how samples were allocated into experimental groups (in the case of clinical studies, please specify allocation to treatment method); if randomization was used, please also state if restricted randomization was applied
- Indicate if masking was used during group allocation, data collection and/or data analysis

Please outline where this information can be found within the submission (e.g., sections or figure legends), or explain why this information doesn’t apply to your submission:

All samples that were directly compared to each other were run on the same plate to prevent potential differences between runs whenever possible. For cases where that was not the case (ultracentrifugation and ExoQuick replicates performed on different days), we ran the same number and type of isolation on each plate to facilitate direct comparison.

**Additional data files (“source data”)**

- We encourage you to upload relevant additional data files, such as numerical data that are represented as a graph in a figure, or as a summary table
- Where provided, these should be in the most useful format, and they can be uploaded as “Source data” files linked to a main figure or table
- Include model definition files including the full list of parameters used
- Include code used for data analysis (e.g., R, MatLab)
- Avoid stating that data files are “available upon request”

Please indicate the figures or tables for which source data files have been provided:

We have uploaded the data from the Simoa runs (in protein concentration) used for all figures in the supplemental “Data.xlsx” file.
